# The relationship between self-reported substance use and psychiatric symptoms in low-threshold methadone maintenance treatment clients

**DOI:** 10.1186/1477-7517-8-18

**Published:** 2011-07-28

**Authors:** Heather G Fulton, Sean P Barrett, Cindy MacIsaac, Sherry H Stewart

**Affiliations:** 1 Department of Psychology, Dalhousie University, Halifax, Nova Scotia, Canada; 2 Department of Psychiatry, Dalhousie University, Halifax, Nova Scotia, Canada; 3 Direction 180, Halifax, Nova Scotia, Canada

**Keywords:** methadone, psychiatric symptoms, psychopathology, low-threshold, substance use, benzodiazepine

## Abstract

**Background:**

Ongoing psychiatric symptoms and substance use are common difficulties experienced by clients enrolled in methadone maintenance treatment (MMT). However, little research to date has evaluated if specific types of current substance use are related to specific types of current psychiatric symptoms. The present study investigated these relationships with a sample of clients enrolled in a low-threshold MMT program (i.e., clients are not expelled if they continue to use substances). Some clients enrolled in low-threshold programs may never achieve complete abstinence from all substances. Thus, understanding the possibly perpetuating relationships between concurrent substance use and psychiatric symptoms is important. Understanding such relationships may aid in developing possible target areas of treatment to reduce substance use and/or related harms in this population.

**Methods:**

Seventy-seven individuals were interviewed regarding methadone usage and current and past substance use. Current psychiatric symptoms were assessed using a modified version of the Psychiatric Diagnostic Screening Questionnaire (PDSQ). Relationships between types of substances used in the past 30 days and the types and number of psychiatric symptoms experienced in the same timeframe were examined.

**Results:**

The majority of participants (87.0%) reported using alcohol, illicit substances, non-prescribed prescription opioids, or non-prescribed benzodiazepines in the past 30 days and 77.9% of participants reported currently experiencing psychiatric symptoms at levels that would likely warrant diagnosis. Current non-prescribed benzodiazepine use was a predictor for increased severity (i.e., symptom count) of almost all anxiety and mood disorders assessed. Conversely, number and presence of generalized anxiety symptoms and presence of social phobia symptoms predicted current non-prescribed benzodiazepine and alcohol use, respectively.

**Conclusions:**

Individuals enrolled in the present low-threshold MMT program experience a wide variety of psychiatric symptoms and continue to use a variety of substances, including opioids. There was a particularly consistent pattern of associations between non-prescribed benzodiazepine use and a variety of psychiatric symptoms (particularly anxiety) suggesting that addressing concurrent illicit benzodiazepine use and anxiety symptoms in MMT clients warrants further clinical attention and research.

## Background

Individuals enrolled in Methadone Maintenance Treatment (MMT) programs often continue to misuse substances [1,2]. Continued use of substances while in MMT is a predictor of poorer MMT treatment outcome [e.g., [3,4]], and represents an ongoing challenge to treatment providers [1,5]. Another important factor related to MMT success is clients' mental health [6,7]. While figures greatly vary, it has been estimated that between 28-76% of MMT clients have at least one co-morbid psychiatric disorder [8-10]. Current psychiatric co-morbidity in MMT clients is associated with poorer psychosocial [11] and medical [12] status as well as decreased quality of life [13]. Similarly, psychiatric distress/severity is generally predictive of poorer MMT outcome [1] although this finding has not always been consistent [10,14]. Current psychiatric symptoms also appear to be associated with ongoing substance use and substance-related problems during MMT. Individuals in MMT with a co-morbid psychiatric disorder have a significantly greater number of lifetime substance use disorders [15], more severe substance use problems [11], and use more substances during MMT [10,16].

While some studies have examined relations between psychiatric symptoms and substance use by MMT clients, most research has focused on presence/absence of any psychiatric co-morbidity (i.e., presence/absence of *any *psychiatric disorder, not presence/absence of *specific *psychiatric disorders), general level of psychiatric distress/severity, or only a limited number of disorders (e.g., depression only). Little research has focused on how different types of psychiatric symptoms may vary by types of substances used. Theory [e.g. [17]] and previous research in non-MMT substance-using samples suggest that specific forms of co-morbidity may be associated with use of specific substances. For example, individuals who fear anxiety-related sensations are more likely to use anxiolytics and to suffer from anxiety-related disorders. Conversely, individuals who tend to act impulsively are more likely to use substances such as cocaine and to suffer psychiatric symptoms in the impulsive domain [18].

The relationships of specific types of self-reported, current substance use to specific types of current psychiatric symptoms were examined in the present study. While evaluating concurrent substance use and psychiatric symptoms in the present study does not permit an analysis of which disorder came first (i.e., a determination of temporality as it may relate to causality), the present evaluation is important to understanding possible perpetuating factors that may maintain both substance use and psychiatric distress in MMT clients. For example, if illicit benzodiazepine use is associated with only one type of psychiatric symptoms (e.g., panic symptoms but not depression), tailoring interventions specific to helping clients cope with panic symptoms could potentially assist in reducing benzodiazepine use and associated overdose risks [19,20]. Further, evaluating concurrent substance use and psychiatric symptom relationships in low-threshold MMT programs (i.e., clients are not expelled if they continue to use substances) is of particular importance given some clients in these programs may never achieve complete abstinence from all substances. Whether clients' psychiatric symptoms are the pathogenic result of substance use or reflect an independent psychiatric disorder may be relatively unimportant if the substance use never ceases. Instead, reducing harms associated with their use (e.g., overdose risk), including reducing distress (e.g., through decreasing anxiety), are important and relevant treatment goals.

In the present study, individuals enrolled in a low-threshold MMT program, who were predominantly receiving treatment for prescription opioid misuse, underwent confidential face-to-face interviews as part of a larger study examining substance use behaviours. It was predicted that current types of substance use would be related to current types of psychiatric symptoms. Specifically it was predicted that anxiety-related symptoms (e.g., symptoms of Generalized Anxiety Disorder [GAD], Post-Traumatic Stress Disorder [PTSD]) would be related to current anxiolytic (e.g., benzodiazepines, alcohol) use. Similarly, it was predicted that impulsive-type psychiatric symptoms (e.g., symptoms related to binge eating) would be related to current stimulant use (e.g., cocaine).

## Methods

### Participants

Seventy-seven participants recruited from a low-threshold MMT program in Halifax, Nova Scotia, Canada took part in the present study. In comparison to more traditional, or "high-threshold", MMT clinics, "low-threshold" clinics do not require clients to be abstinent from all substances in order to remain in treatment [21]. Instead a harm-reduction approach is taken whereby clients obtain privileges, such as the ability to receive their methadone at a community pharmacy, for remaining abstinent from substances. The target population of the clinic are injection drug users who have significant comorbid mental health issues, are dependent on a variety substances, are HIV-, Hepatitis B- and/or C-infected, are homeless and/or street-involved, and/or have been unsuccessful in higher-threshold or abstinence-based treatment programs. All clients enrolled in the MMT program were eligible to participate; there were no exclusion criteria. Demographic data are displayed in Table 1.

**Table 1:** Demographic information reported by sample participants (n = 77)

Characteristic		% (*n*) of Sample or [*M{SD}*]
Age	Years	[39.66{8.79}]
Gender	Male	62.3 (48)
	Female	37.7 (29)
Psychiatric Medication	Prescribed antidepressant (e.g., citalopram)	33.8 (26)
	Prescribed antipsychotic (e.g., quetiapine)	22.1 (17)
	Prescribed any psychiatric medication	63.6 (49)
Education	Less than high school/equivalent	50.6 (39)
	Completed high school/equivalent	49.4 (38)
Ethnicity	Caucasian	80.5 (62)
	Non-Caucasian/multiple ethnicities	19.5 (15)
Income	$10 000 or less per year	67.5 (52)
	More than $10 000 per year	32.5 (25)
Living Status	Renting	87.0 (67)
	Community Shelter	10.4 (8)
	Other	2.6 (2)
Current MMT program use	Years enrolled in current program prior to study interview	[3.00]^1^
	Daily methadone dose (mg)	[112.04{43.97}]
	Days/past 30 methadone used	[28.36{4.48}]
	Proportion enrolled in previous MMT programs	46.8 (36)

^1^Median is reported due to the large standard deviation for this variable: *M*(*SD*) = 3.40(3.05)

### Measures

All measures were administered verbally to participants so that no participant was excluded due to low literacy. Using a semi-structured interview, participants were interviewed regarding demographics, methadone treatment (see Table 1), and current and lifetime substance use [22]. For 19 different substances (see Table 2), participants were asked whether they ever used the substance, age of first use, and number of days in the past 30 they used the substance. Participants were also asked whether they used medications from the classes of prescription opioids (excluding methadone) and benzodiazepines with and without a prescription in their lifetime and in the previous 30 days. Participants who had used *any *benzodiazepines or prescription opioids without a prescription in the past 30 days were defined as "any non-prescribed users". Participants who had *only *used benzodiazepines or prescription opioids with a prescription were defined as "only prescribed users".

**Table 2:** Substance use by sample participants (n = 77) attending a low-threshold MMT program

Drug	% (*n*) Sample Ever Used	Mean Age (*SD*) of First Use for Lifetime Users	% (*n*) Sample Using in Preceding 30 days	Of Lifetime Users, Number of Days of Use in Preceding 30 Days *M*(*SD*)
Tobacco	100.0 (77)	11.5(4.0)	97.4 (75)	29.68(1.95)
Alcohol	98.7 (76)	13.0(4.5)	21.1 (16)	1.72(5.23)
Crack Cocaine	93.5 (72)	26.1(9.1)	44.2 (34)	4.92(9.39)
Powder Cocaine	89.6 (69)	19.2(5.8)	9.1 (7)	0.59(3.65)
Amphetamine/Methamphetamine	57.1 (44)	20.4(7.0)	0 (0)	0(0)
MDMA^1^	66.2 (51)	26.5(9.9)	3.9 (3)	0.12(0.52)
Cannabis	94.8 (73)	13.2(3.4)	48.1 (37)	6.47(10.70)
LSD^2^	84.4 (65)	16.2(3.7)	1.3 (1)	0.14(1.13)
Psilocybin	71.4 (55)	17.0(4.4)	0 (0)	0(0)
Mescaline	59.7 (46)	19.1(4.7)	0 (0)	0(0)
Peyote	14.3 (11)	18.5(2.8)	0 (0)	0(0)
Salvia	14.3 (11)	32.2(11.3)	1.3 (1)	0.90(0.30)
GHB^3^	9.1 (7)	20.4(6.3)	0 (0)	0(0)
Peyote	14.3 (11)	18.5(2.8)	0 (0)	0(0)
PCP^4^	44.2 (34)	20.8(7.4)	1.3 (1)	0.47(2.65)
Ketamine	23.4 (18)	26.1(7.3)	2.6 (2)	1.06(4.24)
Inhalants	40.3 (31)	16.6(7.0)	1.3 (1)	0.32(0.18)
Opium	22.1 (17)	22.4(6.7)	0 (0)	0(0)
Heroin	49.4 (38)	24.3(6.1)	0 (0)	0(0)
Only Prescribed Prescription Opioids	88.3(68)	--^5^	9.1(7)	--
Any Non-prescribed Prescription Opioids	98.7 (76)	--	24.7 (19)	--
Only Prescribed Benzodiazepines	76.6 (59)	--	20.8 (16)	--
Any Non-prescribed Benzodiazepines	89.6 (69)	--	40.3 (31)	--

^1^3,4-Methylenedioxymethamphetamine

^2^Lysergic acid diethylamide

^3^Gamma-hydroxybutyrate

^4^Phencyclidine

^5^Data for the general categories of Only Prescription Opioids, Any Non-Prescribed Prescription Opioids, Only Prescribed Benzodiazepines and Any Non-Prescribed Benzodiazepines were not collected for age of ever use and number days of use/past 30.

For the last 21 participants tested, the above substance use questions were administered a second time by a different interviewer the following day to determine reliability. Substantial reliability for presence of past 30 day use was obtained (Cohen's *κ*s = 0.82-1.00; 95.0-100.0% agreement).

To assess current psychiatric symptoms, a modified Psychiatric Diagnostic Screening Questionnaire (PDSQ [23]) was used. This measure contained 125 yes/no questions regarding experiencing symptoms of 13 DSM-IV [24] Axis I disorders in the past two weeks or past 30 days (past two weeks and past six months are used in the original version). This modification enabled the period of reported psychiatric symptoms to be within the substance use interview's assessment of use in the preceding 30 days. An individual screened positive for a disorder on the PDSQ if s/he endorsed the predetermined minimal number of symptoms for that diagnostic category (see Table 3). Screening positive for a disorder on the PDSQ suggests that an individual would be significantly more likely to qualify for a diagnosis of that disorder than someone who did not screen positive [23]. In previous studies the PDSQ has been found to have good sensitivity (90% of cases screening positive warranted a diagnosis), negative predictive values (97% of cases that did not screen positive did not warrant a diagnosis), reliable and valid (see [23] for review) - even in a sample of individuals with substance use disorders [25].

**Table 3:** Psychiatric symptoms of sample (n = 77) as assessed by the PDSQ

Disorder (# of symptoms assessed on PDSQ; # of symptoms required for a positive screen)	Mean(*SD*) number of symptoms endorsed by sample	% Sample Screening Positive for Disorder(*n*)
Eating Disorder (10;7)	1.64 (2.55)	7.8 (6)
Psychosis (6;1)	0.68 (1.24)	32.5 (25)
Hypochondriasis (5;1)	0.75 (1.31)	33.8 (26)
Somatization Disorder (5;1)	1.36 (1.38)	42.9 (33)
Depression (21;9)	6.45 (5.20)	31.2 (24)
Post Traumatic Stress Disorder ([PTSD] 15;5)	5.78 (5.26)	48.1 (37)
Obsessive Compulsive Disorder ([OCD] 7;1)	1.58 (2.10)	49.4 (38)
Panic Disorder (8;4)	2.05 (2.49)	27.3 (21)
Agoraphobia (12;4)	2.08 (2.83)	24.7 (19)
Social Phobia (15;4)	2.65 (3.80)	29.9 (23)
Generalized Anxiety Disorder ([GAD] 10;7)	3.81 (3.77)	28.6 (22)
Total number of symptoms endorsed (114)	28.83 (24.00)	

The modified and original versions of the PDSQ were administered to the last 21 participants in the present study by separate interviewers one day apart. Good reliability between the two versions in terms of the number of symptoms endorsed and number of positive screens of disorders was found (*r*s = .87,.81, respectively).

Questions relating to drug and alcohol dependence were excluded from analysis given the present study's objective of evaluating the relationship between substance use and symptoms of psychiatric disorders other than substance use disorders.

### Procedure

All clients enrolled in the MMT program were informed of their eligibility to participate in the present study. Clients were informed that all study information would be kept confidential, participation (or lack thereof) would not affect their treatment, and participation was voluntary. All interviews were conducted by personnel separate from clinic staff in a private room at the clinic. Participants gave verbal and written informed consent and were compensated $20 at the completion of the study. All sampling, procedures, and materials were reviewed and approved by the Dalhousie and Capital Health Research Ethics Boards.

### Analyses

In order to ensure adequate variability for statistical analyses, if at least 10% of the sample, but not more than 90%, had used a substance in the past 30 days or screened positive for a psychiatric disorder, the variable was included in further analyses examining the relationships between current substance use and psychiatric symptoms. Dichotomous variables were analyzed using chi-square (χ^2^) tests; two-sided Fisher's exact tests were used whenever expected counts were less than 5 to minimize chances of Type 1 error [26]. Continuous variables were analyzed using independent sample t-tests and bivariate correlations.

Because substances are often used in a polysubstance context [27], multiple regressions were conducted to evaluate whether current use of specific substance(s) was(were) better predictor(s) of the number of psychiatric symptoms endorsed for each type of disorder assessed by the PDSQ. Logistic regressions were also conducted to evaluate whether current use of specific substance(s) was(were) better predictor(s) of screening positive on the PDSQ for different types of psychiatric symptoms.

Because psychiatric symptoms also often co-occur [28], and due to the possible bidirectional relationship of psychiatric symptoms and substance use [29], logistic regressions were conducted to evaluate if the number of specific types of psychiatric symptoms were better predictors of the likelihood to be currently using different substances. Additional logistic regressions were conducted to evaluate whether screening positive for certain types of psychiatric symptoms on the PDSQ would also predict the likelihood of currently using different substances.

## Results

### Substance use

The majority of participants (87.0%, *n *= 67/77) reported using alcohol, illicit substances, non-prescribed prescription opioids, and/or non-prescribed benzodiazepines at least once in the past 30 days (see Table 2). Participants used, on average, 2.04 (*SD *= 1.67) different substances, excluding tobacco, in the past 30 days; or 3.01 (*SD *= 1.68) different substances if tobacco is included. Prescription opioids and benzodiazepines were each counted as one substance, regardless of whether the type of medication was used with and/or without a prescription. Participants reported that all current benzodiazepine and opioid prescriptions were from doctors not affiliated with the present MMT program; prescriptions were obtained from family or emergency room doctors.

### Psychiatric Symptoms

Sixty participants (77.9%, *n *= 60/77) screened positive for at least one psychiatric disorder on the modified PDSQ. Participants, on average, screened positive for 3.52(*SD *= 3.16) different psychiatric disorders (see Table 3).

Because reporting of psychiatric symptoms has been found to decrease with time enrolled in MMT in some studies [29,30], relationships between psychiatric symptoms and current methadone treatment variables were examined. There were no significant differences in current methadone dose or duration enrolled in the current MMT program between those who did and did not screen positive for any psychiatric disorders (*p*s > 0.05).

### Current Substance Use and Psychiatric Symptoms

Results of the multiple regression analyses of current substance use predicting the number of symptoms of different types of psychiatric disorders are displayed in Table 4. Non-prescribed benzodiazepine use significantly predicted the number of symptoms endorsed for almost all mood and anxiety symptoms assessed as well as the total number of symptoms endorsed on the PDSQ. Similar results were also obtained when logistic regressions were run with current substance use predicting the likelihood to screen positive on the PDSQ for different disorders (see Table 5). Non-prescribed benzodiazepine use significantly predicted the likelihood to screen positive for depression, PTSD, GAD, social phobia, as well as the likelihood to screen positive for at least one disorder on the PDSQ (Any disorder assessed on the PDSQ). Current alcohol use was also a significant univariate predictor for likelihood to screen positive for social phobia.

**Table 4:** Multiple regressions of past 30 day substance-use predicting psychiatric symptoms

Dependent Variable (# of symptoms endorsed on PDSQ)	Model *F*(6,67) =	*p*	Adjusted *R*^2^	Significant predictors (*p*)	Beta
Psychosis	1.44	.212	.04		
Hypochondriasis	1.71	.133	.06		
Somatization disorder	1.83	.107	.06		
Depression	6.16	<.001	.27	-Any non-prescribed benzodiazepine use (<.001)	.58
PTSD	3.60	.004	.18	-Any non-prescribed benzodiazepine use (<.001)	.46
OCD	3.99	.002	.20	-Any non-prescribed benzodiazepine use (<.001)	.44
Panic disorder	2.42	.035	.11	-Any non-prescribed benzodiazepine use (.001)	.41
Agoraphobia	1.40	.226	.03		
Social phobia	2.73	.020	.12	-Any non-prescribed benzodiazepine use (.004)	.37
GAD	6.06	<.001	.29	-Any non-prescribed benzodiazepine use (<.001)	.60
Total number of all symptoms assessed	3.08	.010	.15	-Any non-prescribed benzodiazepine use (<.001)	.49

*Predictors entered in all regressions: Any non-prescribed benzodiazepine use, Only prescribed benzodiazepine use, Any non-prescribed prescription opioid use, Any alcohol use, Any cannabis use, and Any crack use in the past 30 days.

**Significant univariate predictors are presented only in the case of a significant multivariate model.

**Table 5:** Logistic regressions of past 30 day substance use predicting screening positive for types of psychiatric symptoms

Dependent Variable (screening positive for disorder on PDSQ)	Model *X*^*2*^(*p*)	Significant predictors (*p*)	Odds ratio (OR)	95% confidence interval for OR
Psychosis	8.01(.238)			
Hypochondriasis	10.08(.121)			
Somatization disorder	10.50(.105)			
Depression	22.08(.001)	-Any non-prescribed benzodiazepine use (.001)	9.63	2.67-34.68
PTSD	22.40(.001)	-Any non-prescribed benzodiazepine use (.001)	7.56	2.26-25.31
OCD	11.91(.064)			
Panic disorder	10.43(.108)			
Agoraphobia	10.82(.091)			
Social phobia	23.37(.001)	-Any non-prescribed benzodiazepine use (.003)	7.70	2.00-29.58
		-Alcohol use (.018)	6.59	1.28-31.33
GAD	21.06(.002)	-Any non-prescribed benzodiazepine use (<.001)	12.30	3.17-47.72
Any disorder assessed on PDSQ	18.25(.006)	-Any non-prescribed benzodiazepine use (.006)	22.14	2.42-203.00

*Predictors entered in all regressions: Any non-prescribed benzodiazepine use, Only prescribed benzodiazepine use, Any non-prescribed prescription opioid use, Any alcohol use, Any cannabis use, and Any crack use in the past 30 days.

**Significant univariate predictors are presented only in the case of a significant multivariate model.

For the logistic regressions of psychiatric symptoms predicting the likelihood to use different substances, non-prescribed benzodiazepine use was significantly predicted by the number of different types of psychiatric symptoms (χ^2^(10) = 36.27, *p *< .001); number of GAD symptoms was the only univariate predictor (*p *= .024, OR = 1.48, 95%CI = 1.05-2.08). When screening positive for different psychiatric disorders were used as predictors in the regression analyses instead of the number of psychiatric symptoms endorsed, current non-prescribed benzodiazepine use was significantly predicted (χ^2^(10) = 35.24, *p *< .001) by screening positive for GAD (*p *= .033, OR = 17.52, 95%CI = 1.26-246.10) and agoraphobia (*p *= .040, OR = 0.07, 95%CI = 0.01-0.88). That is, screening positive for GAD was associated with an *increased *likelihood of currently using non-prescribed benzodiazepines while screening positive for agoraphobia was associated with a *decreased *likelihood of currently using non-prescribed benzodiazepines. However, the relationship of screening positive for agoraphobia and past 30 day non-prescribed benzodiazepine use was examined further for possible suppressor effects. There was no significant correlation between screening positive for agoraphobia and past 30 day non-prescribed benzodiazepine use (point biserial *r *= .13, *p *= .277) but screening positive for GAD and agoraphobia were highly correlated (point biserial *r *= .57, *p *< .001). Thus, it is likely that the relationship between agoraphobia and non-prescribed benzodiazepine use reflects a suppressor effect [31]^a^. Lastly, past 30 day alcohol use was found to be significantly predicted (χ^2^(10) = 18.97, *p *= .041) by screening positive for social phobia (*p *= .025, OR = 15.28, 95%CI = 1.40-166.56).

## Discussion

The present study found high rates of current use of a variety of substances as reported by clients enrolled in a low-threshold MMT program. Similar to previous studies of substance use by MMT clients [1,2], alcohol, cannabis, cocaine, prescription opioids, and benzodiazepines were commonly-used substances; current use of hallucinogens or inhalants was rare. Consistent with previous research in higher-threshold MMT programs (e.g., [9,10,12]), the present study also found high rates of psychiatric symptom reporting by low-threshold MMT clients. For many clients, these reports revealed levels of psychiatric symptoms that may warrant clinical diagnosis [23].

As expected, we found support for relations between current substance use and current psychiatric symptom reporting. In particular, current non-prescribed benzodiazepine use predicted the number of psychiatric symptoms endorsed for most mood- and anxiety-related psychiatric disorders as well as predicting the likelihood of screening positive for most mood- and anxiety-related disorders assessed on the PDSQ. That is, current non-prescribed benzodiazepine use was associated with an increased number of psychiatric symptoms, and was associated with an increased likelihood of experiencing different psychiatric symptoms at levels that may warrant diagnosis. Current alcohol use (in addition to non-prescribed benzodiazepine use) was also found to be associated with an increased likelihood to screen positive for social phobia (see Table 5).

Conversely, current psychiatric symptoms were found to predict the likelihood of different types of current substance use. Number of GAD symptoms, as well as screening positive for this disorder on the PDSQ, made a unique contribution in predicting current non-prescribed benzodiazepine use. Screening positive for social phobia (but not the number of these types of symptoms) was associated with an increased likelihood of current alcohol use.

The findings that any current non-prescribed benzodiazepine use uniquely predicted number and the likelihood of experiencing psychiatric symptoms-namely anxiety and depression, and that GAD symptoms appear to be a unique predictor among psychiatric symptoms of current non-prescribed benzodiazepine use, is consistent with previous literature. While little research has indicated whether benzodiazepine use was prescribed or non-prescribed, benzodiazepine users in MMT programs have been found to have higher levels of anxiety symptoms [32,33], suicidal ideation, more suicide attempts [34] and lower psychosocial functioning [33] than non-users.

It is possible that non-prescribed benzodiazepines are being used to self medicate distressing psychiatric symptoms such as generalized anxiety symptoms [17]. It is also possible anxiety-related withdrawal symptoms from benzodiazepines may be causing or exacerbating any existing anxiety symptoms [19,35-37]. Alternatively, these individuals could have a common underlying vulnerability to both benzodiazepine use and psychiatric symptoms [30,38]. Regardless of the basis for the relationship, these findings, in combination with existing literature [32,33], suggest that non-prescribed benzodiazepine use may be indicative of higher levels of psychopathology and related problems in MMT clients. Multiple systemic barriers often prevent individuals with concurrent psychiatric and substance use issues from accessing appropriate treatment (e.g., organization of services, finances [39]). Thus, further investigations and treatment development in this area are likely to be fruitful.

Of note is the finding that screening positive for agoraphobia was a significant predictor of *decreased *likelihood to use non-prescribed benzodiazepines and this relationship was likely indicative of a suppressor effect [31]. In this case, while screening positive for GAD may capture much of the variance in predicting non-prescribed benzodiazepine use, it is likely that screening positive for agoraphobia improves prediction of non-prescribed benzodiazepine use (despite the lack of an independent relationship with between these two variables) by accounting for avoidance related to anxiety. That is, agoraphobia may be protective of non-prescribed benzodiazepine use because individuals who often avoid anxiety-inducing situations may not feel they need to use benzodiazepines to manage their anxiety. They may be able to avoid anxiety through avoiding certain situations, whereas people with GAD symptoms may avoid anxiety through non-prescribed benzodiazepine use. Further investigations are needed to determine if this hypothesis is correct.

The finding that social phobia and alcohol use were related is also consistent with previous literature. Many studies have found high rates of comorbidity between social anxiety and alcohol problems [see [40] for review]. Alcohol use while enrolled in MMT is also associated with increased overdose risk [20]. Thus, programming to address this association could also be beneficial to decreasing mortality and other harms.

There are a number of limitations to the present study. First, the present study consisted of a relatively small sample. Thus, there could be concerns regarding reliability of the findings. However, Type II error, not Type I, is more likely with small sample sizes, and the present sample size exceeds the 5:1 (participants: predictor variable) regression guidelines, as well as those suggested by Miles and Shevlin (2001) [26]. Second, the PDSQ is weighted for emphasis on anxiety disorders. Although it assesses eating disorder symptoms, the PDSQ does not assess other disorders that theoretically would be related to stimulant use rather than benzodiazepine use (e.g., ADHD). More complex relationships between types of current psychiatric symptoms and types of current substance use may be revealed with more comprehensive psychiatric assessments. Third, the PDSQ does not assess Axis II [24] symptomatology. Personality disorders, particularly antisocial and borderline, have been found to be highly prevalent in opioid-dependent individuals (see [34] for review; prevalences can vary between 15-73% for presence of any personality disorder compared to 10% in the general population). Opioid-dependent individuals with such disorders have been found to have increased severity of depression, anxiety and substance use problems (e.g., alcohol dependence) [41]. Further investigations into the extent to which such personality pathology may be accounting for the observed psychiatric symptoms and substance use relationships are warranted. Fourth, it is possible that the positive screens on the PDSQ may be over inclusive for some disorders regarding likelihood to receive a diagnosis. It seems somewhat unlikely that almost 50% of the sample would receive a legitimate diagnosis of OCD or somatization disorder if further assessed. Instead, endorsing items such as repeated checking of locks on doors may better reflect the sometimes unstable and unsafe circumstances of participants' housing, and endorsement of somatization disorder symptoms such as "stomach and intestinal problems" may reflect side effects of MMT. Despite these possibilities, the rates of psychiatric symptoms reporting in the present study were comparable to previous research with methadone clients (e.g., [9,10,12]) and the PDSQ clearly measured some level of specific psychiatric symptomatology in the present study given the large effect sizes and consistency of relationships with non-prescribed benzodiazepine use. Another limitation of the present study was that substance use behavior was based on self-report. While there are some criticisms of this method [42], it has been found to produce accurate results, particularly under circumstances enhancing accurate reporting like those used in the present study [42,43]. Because of assurances of confidentiality, participation not influencing treatment, and compensation for participation not being contingent upon reporting (or not reporting) substance use, there was no motivation to minimize or exaggerate any substance use. Indeed, when assessed, the test-retest reliability of the present study measures was excellent.

The present research has a number of implications for both further practice and research. In terms of practice, it was somewhat surprising to have found a relatively large (20.8%, *n *= 16/77) percentage of clients having current prescriptions for benzodiazepines and/or opioids from health professionals outside of the current MMT program. It is possible some clients' family or emergency room doctors may not be aware their clients are on methadone, and/or MMT programs may not be aware a client is obtaining benzodiazepines or opioids via other medical professionals. This suggests that access to updated health records- for both MMT programs and other physicians (e.g., through electronic health records) could be beneficial given prescription drug monitoring programs may not always flag occurrences such as those in the present study. While prescribed benzodiazepine use did not have the same associations as non-prescribed use, there is still substantial overdose risk by concurrently using benzodiazepines with methadone [20,44]. In terms of research implications, given that there is remaining uncertainty if individuals enrolled in MMT may be using non-prescribed benzodiazepines to manage distressing mood states (such as anxiety), or if the reverse or another reason may be accounting for the observed relationships, research examining longitudinal patterns of substance use and psychiatric symptoms in MMT clients, or specific occasions of use should be conducted to further examine these competing hypotheses. The present study findings also suggest that future research and practice could focus on further developing, tailoring, and evaluating interventions to address benzodiazepine use by MMT clients. Possible therapeutic targets could include tailored interventions focusing on managing generalized anxiety symptoms and psychoeducation (e.g., [45]) regarding the biological and psychological effects (both long- and short-term) of using benzodiazepines. It is possible such interventions could help this population to reduce benzodiazepine use, its related negative effects, as well as associated psychiatric symptom severity. Similar tailored programming may also be beneficial if focused on alcohol use and social anxiety symptoms. Previous research suggests that addressing both psychiatric symptoms and substance use concurrently in treatment, in an integrated fashion, is likely to be the most favorable treatment approach [46].

## Conclusions

Low-threshold MMT clients report high rates of both current substance use and current psychiatric symptoms. Non-prescribed benzodiazepine use appears to be a unique predictor of experiencing psychiatric symptoms- particularly various types of anxiety. Conversely, GAD symptoms appear to be a unique predictor amongst psychiatric symptoms in identifying current non-prescribed benzodiazepine use. Further investigations regarding the temporal nature of benzodiazepine use and psychiatric symptoms, as well as possible development of interventions tailored specifically to addressing this relationship, could be beneficial to our understanding of psychopathology and substance use. Further, additional research and clinical work in this area may assist in reducing the serious risk of overdose and harm posed by using substances, particularly benzodiazepines, while enrolled in MMT.

## Endnotes

^a^Briefly, a classic suppressor effect occurs when the addition of a predictor to the regression results in another predictor (or group of predictors) increasing in predictive validity, even though the newly added predictor may be unrelated to the dependent variable. See [31] for further explanation.

## List of abbreviations

**CI**: Confidence Interval; **F**: F ratio for overall regression model; **GAD**: Generalized Anxiety Disorder; **GHB**: Gamma-hydroxybutyrate; **LSD**: Lysergic acid diethylamide; ***M: ***Mean; **MDMA**: 3,4-Methylenedioxymethamphetamine; **MMT**: Methadone Maintenance Treatment; ***n: ***Number of participants in subsample; **OCD**: Obsessive Compulsive Disorder; **OR**: Odds Ratio; ***p***: Probability of Type 1 error; **PCP**: Phencyclidine; **PDSQ**: Psychiatric Diagnostic Screening Questionnaire; **PTSD**: Post-Traumatic Stress Disorder; ***r***: Correlation coefficient; **R**^**2**^: Coefficient of determination; ***SD***: Standard Deviation; **χ**^**2**^: Chi square.

## Competing interests

The authors declare that they have no competing interests.

## Authors' contributions

All authors have contributed significantly to this research report and have read and approved the final manuscript. HGF assisted with the planning of the study, conducted data collection, contributed to data analysis, interpretation, write-up and funding of the study. SPB and SHS assisted with planning of the study, data analysis, interpretation of results, writing of the manuscript and funding of the study. CM assisted with planning of the study, data collection, practice implications, as well as reviewed and revised the drafts of the manuscript.
